# Equivalent intraperitoneal doses of ibuprofen supplemented in drinking water or in diet: a behavioral and biochemical assay using antinociceptive and thromboxane inhibitory dose–response curves in mice

**DOI:** 10.7717/peerj.2239

**Published:** 2016-07-19

**Authors:** Raghda A.M. Salama, Nesreen H. El Gayar, Sonia S. Georgy, May Hamza

**Affiliations:** Department of Pharmacology, Faculty of Medicine, Ain Shams University, Cairo, Egypt

**Keywords:** Drug administration routes, Formalin test, Dose–response relationship, Ibuprofen, Thromboxane B_2_, Incisional pain

## Abstract

**Background.** Ibuprofen is used chronically in different animal models of inflammation by administration in drinking water or in diet due to its short half-life. Though this practice has been used for years, ibuprofen doses were never assayed against parenteral dose–response curves. This study aims at identifying the equivalent intraperitoneal (i.p.) doses of ibuprofen, when it is administered in drinking water or in diet.

**Methods.** Bioassays were performed using formalin test and incisional pain model for antinociceptive efficacy and serum TXB_2_ for eicosanoid inhibitory activity. The dose–response curve of i.p. administered ibuprofen was constructed for each test using 50, 75, 100 and 200 mg/kg body weight (b.w.). The dose–response curves were constructed of phase 2a of the formalin test (the most sensitive phase to COX inhibitory agents), the area under the ‘change in mechanical threshold’-time curve in the incisional pain model and serum TXB_2_ levels. The assayed ibuprofen concentrations administered in drinking water were 0.2, 0.35, 0.6 mg/ml and those administered in diet were 82, 263, 375 mg/kg diet.

**Results.** The 3 concentrations applied in drinking water lay between 73.6 and 85.5 mg/kg b.w., i.p., in case of the formalin test; between 58.9 and 77.8 mg/kg b.w., i.p., in case of the incisional pain model; and between 71.8 and 125.8 mg/kg b.w., i.p., in case of serum TXB_2_ levels. The 3 concentrations administered in diet lay between 67.6 and 83.8 mg/kg b.w., i.p., in case of the formalin test; between 52.7 and 68.6 mg/kg b.w., i.p., in case of the incisional pain model; and between 63.6 and 92.5 mg/kg b.w., i.p., in case of serum TXB_2_ levels.

**Discussion.** The increment in pharmacological effects of different doses of continuously administered ibuprofen in drinking water or diet do not parallel those of i.p. administered ibuprofen. It is therefore difficult to assume the equivalent parenteral daily doses based on mathematical calculations.

## Introduction

Ibuprofen is a non-steroidal anti-inflammatory drug. It is one of the most widely used analgesics both acutely and chronically (Rainsford, 2009). In experimental animals, it is used chronically in models of arthritis (De Vries et al., 1988), radiation pneumonitis (Gross, Holloway & Narine, 1991) and Alzheimer’s disease (Dong et al., 2014).

Ibuprofen has a short half-life (2.5 h; Rainsford, 2009) and therefore requires continuous administration in cases of chronic regimens, in experimental animals. For this purpose, it has been reportedly administrated in drinking water or supplemented in chow in several studies. For example, mice were administered ibuprofen in drinking water in a concentration of 1 mg/ml for 7 days for acute colitis (Matos et al., 2013) and in concentrations of 0.2 and 0.6 mg/ml for 25 days to prevent spontaneous lung metastases secondary to mammary carcinoma (Khoo et al., 1992). It was also administered in a concentration of 0.35 mg/ml for 2 weeks to study possible beneficial effect on cardiac functions following cardiac injury (Kofidis et al., 2006). The concentration of 0.2 mg/ml was also used for 7 days in a postsurgical pain model (Hayes et al., 2000) and for 14 days in BCG inoculated mice to assess its possible antidepressant effect (Saleh et al., 2014).

Ibuprofen was also administered in food as ibuprofen-supplemented chow in a mouse model of Alzheimer’s disease in a dose of 375 mg/kg diet, either for 6 weeks (Yan et al., 2003), two months (DiBattista et al., 2016) or for 6 months (Dong et al., 2014). It was also given in a dose of 263 mg/kg diet for 25 weeks to inhibit lung tumorigenesis in mice (Jalbert & Castonguay, 1992). In the same study, doses of 82 and 328 mg/kg diet, administered for 6 weeks, were assessed for GIT toxicity.

Though ibuprofen has been frequently administered in drinking water or supplemented in food, we could only locate a few studies expressing the equivalent daily dose of the concentrations used. These were mathematically calculated, for example Yan et al. (2003) and Hayes et al. (2000). However, when deciding on a dose to be used experimentally, the pharmacodynamic effects of this dose is the defining factor. Therefore, we tried, in the present work, to assess different doses supplemented in drinking water or in diet, through assaying them pharmacodynamically against parenteral dose–response curves.

The intraperitoneal route (i.p.) is the most common route used in small laboratory animals such as mice and rats, being technically simple and easy. It results in fast absorption into the vasculature with a rate of absorption of one-half to one-fourth that of the intravenous route (Shimizu, 2004; Woodard, 1965). Taken together, it is plausible to assay ibuprofen concentrations added to drinking water or supplemented in diet against an i.p. dose–response curve.

Ibuprofen is a non-selective reversible competitive cyclooxygenase (COX) inhibitor (McAdam et al., 1999). Besides COX inhibition, several other mechanisms are involved in ibuprofen’s action (Hamza & Dionne, 2009). The activity of COX-2 (the induced form) increases during inflammatory reactions (Frolich, 1997), and is assumed to play an important role in inflammatory pain models (Vanegas & Schaible, 2001). On the other hand, thromboxane A_2_ (TXA_2_) is a product of COX-1 found in platelets and inhibition of its synthesis inhibits platelet aggregation (De Caterina et al., 1985). Therefore, assessing ibuprofen’s antinociceptive effect in the inflammatory pain models and its inhibitory effect on serum TXA_2_ is assumed to reflect its inhibitory activity on both COX isoforms.

The aim of the present work is to identify the equivalent intraperitoneal dose for ibuprofen supplemented in drinking water or in diet as regards:

1. Its antinociceptive efficacy; using two different inflammatory pain models (the formalin test and the incisional pain model).

2. Its eicosanoid inhibitory activity; using serum thromboxane B_2_ levels (TXB_2_).

## Materials and Methods

### Animals

234 male Swiss albino mice weighing 25–43 grams, bred at the Holding Company for Biological Products & Vaccines (VACCERA, Helwan, Egypt), were used. They were housed up to ten per cage. Food and water were available *ad libitum*. The animals were allowed to acclimatize for 1 week before starting the experiments. The number of mice used was kept to a minimum, and all mice were euthanatized immediately after the experiments. Each mouse was used once. All experiments were carried out between 7:00 am and 5:00 pm. The protocol was approved by the Research Ethics Committee (REC) of the Faculty of Medicine, Ain Shams University, FWA 00017585 (approval number R5/2016) and experiments were conducted in accordance with the ethical guidelines for the study of experimental pain in conscious animals of the International Association of the Study of Pain.

### Ibuprofen administration

Ibuprofen sodium was purchased from Sigma Aldrich Chemical (USA). It was dissolved in saline and administered at doses of 50, 75, 100 and 200 mg/kg, i.p. In another set of experiments, ibuprofen sodium was dissolved in drinking water at concentrations of 0.2, 0.35 and 0.6 mg/ml or mixed in diet at doses of 82, 263 and 375 mg/kg diet. Chow was ground to powder. The calculated dose of ibuprofen was dissolved in water, and mixed with the ground chow to form dough, which was reshaped again. Ibuprofen dissolved in drinking water or mixed with diet was administered for two days before performing the formalin test or the incisional pain model, or blood sampling.

### Formalin test

The formalin test was performed as described earlier (El-Morsy, Aboul-Ela & Hasanin, 2013; Guhring et al., 2002). Each mouse was placed in a glass cage (40 × 30 × 20 cm), with a mirror fixed behind it to allow an unobstructed view of the paws. Mice were habituated to this environment for at least 1 h prior to formalin injection.

50 µl of 3% formalin was injected subcutaneously into the dorsal aspect of the right hindpaw using a 27-gauge needle. Immediately after the formalin injection, mice were placed back in the glass cage and their behavior was observed continuously for the next 60 min. The number of flinches (rapid shaking of the injected paw) was counted and recorded every minute. The time spent licking and/or biting the injected paw was also estimated and recorded every minute. The nociceptive response was expressed as a “pain-related behavior” score, which was calculated as the total number of flinches +1/10 of the time spent in licking and/or biting the injected paw. The formalin test was performed by a single observer, under constant environmental conditions. Each animal was used only once.

### Incisional pain model

#### Surgical procedure

Under light ether anesthesia, a number-11 blade was used to induce a 5-mm longitudinal incision through the skin, fascia, and muscle of the plantar aspect of the right hindpaw. Hemostasis with gentle pressure was applied. Animals were allowed to recover after surgery (Saad et al., 2016; Tillu et al., 2012).

#### Assessing tactile allodynia

Mice were placed in a custom-made elevated wire mesh floor surrounded by a clear glass chamber (10 × 10 × 15 cm). Mice were kept in the chambers for 30 min before starting any assessment. Nine von Frey filaments (Ugo Basile, North America) were applied to the planter aspect of the foot, from the least to greatest forces (0.16, 0.4, 0.6, 1, 1.4, 2, 4, 6 and 8 gram forces). Each monofilament was introduced through the wire mesh and was applied perpendicularly with sufficient force to bend, and a response (ranked either 0, no response or 1, a brisk withdrawal) was observed. Each filament was applied five times to each hindpaw, with 30 s between applications. The response threshold was defined as the lowest force that caused at least 3 withdrawals out of the 5 consecutive applications, i.e., the 60% response threshold (Jiang et al., 2013; Saad et al., 2016; Xie et al., 2005). The procedure was performed for at least 3 days before surgery to accustom the mice to the testing procedures and to achieve a stable baseline record. Immediately after surgery, mice were placed in the chambers and tactile allodynia was assessed at 0.5, 1 and 2 h after incision, where von Frey filaments were applied adjacent to the wound.

### Serum thromboxane B_2_ assay

Blood samples were collected from retro-orbital capillaries of veins. Samples were left at 37 °C for 1 h, after which they were centrifuged at 3,000 rpm for 20 min and the serum supernatant was collected in an Eppendorf tube and stored at −80 °C until analysis. TXA_2_ is very unstable; thus the more stable derivative TXB_2_ was measured in serum instead (Kuwano et al., 2004). The immunoassay kit (Kamiya Biomedical Company, Cat. No. KT-29740) was used and the procedure was performed according to the manufacturer’s instructions.

### Experimental protocol

For the formalin test, 5 groups of mice received either saline or one of the 4 graded doses of ibuprofen i.p. to construct a dose–response curve. Three groups of mice received different concentrations of ibuprofen in drinking water and 3 other groups received ibuprofen mixed in their diet. The number of mice per group was 6, except for the control group, where 8 mice were used.

For testing tactile allodynia following surgical incision, 5 other groups of mice received either saline or one of the 4 graded doses of ibuprofen i.p. to construct a dose–response curve. 3 groups of mice received different concentrations of ibuprofen in drinking water and 3 other groups received ibuprofen mixed in diet. A naïve group was also tested. The number of mice per group was 8 mice.

For thromboxane B_2_ estimation, ibuprofen in the same doses mentioned above was administered to another 11 groups (6 mice, each). Medicated water or food was withdrawn for 2 h, and then blood samples were collected.

### Water consumption in mice

Mice were individually housed for 48 h and water consumption was measured every 24 h. The mean of the two readings was used to calculate weight-corrected average daily water consumption.

### Expression of results and statistical analysis

Statistical analyses were carried out using Graphpad prism (version 5.01, CA, USA). Data are expressed as mean ± S.D. in text and as mean ± SEM in figures. For comparisons among different groups, one-way analysis of variance (ANOVA) followed by Dunnett’s or Bonferroni’s multiple comparison tests were performed. Pearson correlation analysis was used to test for correlation between daily water consumption and body weight. Results were considered significant at *p* < 0.05.

*Regarding formalin test*, the sum of pain-related behavior scores was used for comparisons across groups. The sum of the first 15 min was used to express phase 1, the sum of the next 45 min was used to express phase 2, and the sum of pain-related behavior scores between the 20th and 40th minutes represents phase 2a. Phase 2a was used to construct the dose–response curve.

*To test the effect on tactile allodynia*, the difference in the 60% mechanical threshold between the incised and non-incised paws in different groups, referred to as “change in mechanical threshold”, was estimated at each time point. Decrease in the 60% mechanical threshold in the incised hindpaw and hence significant increase in the change in mechanical threshold was refered to as “tactile allodynia”. Repeated-measure two-way ANOVA was used to compare the changes in mechanical threshold. Comparisons at each time point were performed using Bonferroni posttests. The area under the ‘change in mechanical threshold-time’ curve at the first 2 h (AUC_0–2h_) was used to construct the dose–response curve.

*To construct dose–response curves*, the mean responses to different i.p. doses were plotted as a scatterplot against the logarithm of i.p. doses. The control group (0 mg/kg) was considered as 1; to be transformed to 0 on the logarithmic scale. A sigmoid curve was fitted using Graphpad prism software. The equation used was dose–response; log (inhibitor) vs. response—Variable slope:

\begin{eqnarray*}Y=\mathrm{Bottom}+(\mathrm{Top}-\mathrm{Bottom})/(1+1{0}^{\wedge }((\mathrm{LogIC50}-X)\ast \mathrm{Hill}\hspace*{1em}\mathrm{slope})). \end{eqnarray*}

The equivelent i.p. doses were interpolated by the same software.

In the case of tactile allodynia, 2 mice in each group of ibuprofen supplemented in drinking water and in diet were out of range and thus the equivelent i.p. dose was considered as the nearest calculable point. These individual data were only used to calculate the S.D. for comparison between different tests (Fig. 8).

## Results

### Effect of different doses of ibuprofen injected i.p. on formalin-induced pain-related behavior in mice

Ibuprofen injected i.p. in different doses (50, 75, 100 & 200 mg/kg), 30 min before subcutaneous formalin injection, resulted in a dose-dependent decrease in pain-related behavior.

The dose 300 mg/kg was tried (*n* = 4) but resulted in a very high mortality rate (75%) and the non-fatal incident showed prostration, which did not allow for behavioral testing.

**Figure 1:**
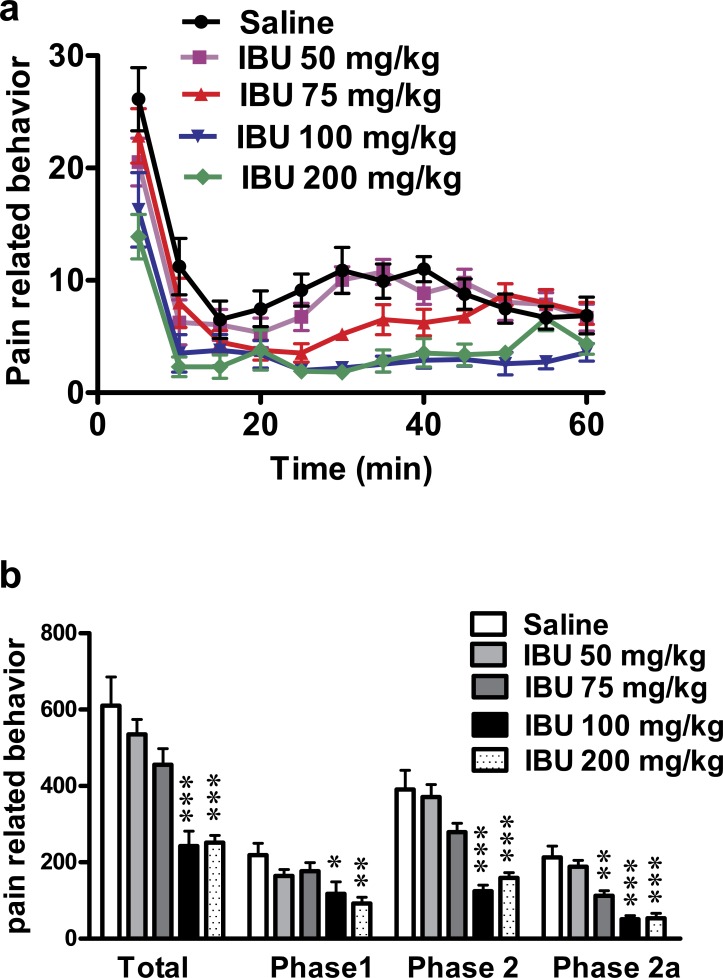
Effect of different doses of ibuprofen, administered i.p., on formalin-induced pain-related behavior in mice. Data are presented as mean ± S.E.M. (A) Each point represents the mean of pain related behavior over 5 min. (B) Each column represents the sum of pain-related behavior. Phase 1 represents the first 15 min of the formalin test. Phase 2 represents the next 45 min. The total is the sum of both phases and phase 2a represents the time between the 20^th^ and the 40^th^ min. ^∗^ indicates *p* < 0.05, ^∗∗^ indicates *p* < 0.01 and ^∗∗∗^ indicates *p* < 0.001, compared to saline injected group (one way ANOVA, followed by Dunnett’s multiple comparison test). Number of animals for each subgroup = 6, except for control (saline) group = 8.

Ibuprofen (200 mg/kg) induced a significant decrease in total pain-related behavior, in phase 1 (*p* < 0.01) and in phase 2, including phase 2a (*p* < 0.001; One-way ANOVA followed by Dunnett’s multiple comparison posttest; Fig. 1). The dose of 100 mg/kg ibuprofen induced a significant decrease in total pain-related behavior, phase 1 and phase 2 including phase 2a (*p* < 0.001 except for phase 1, where *p* < 0.05; One-way ANOVA followed by Dunnett’s multiple comparison posttest). The dose of 75 mg/kg induced a significant decrease only in phase 2a (*p* < 0.01; One-way ANOVA followed by Dunnett’s multiple comparison posttest), while the 50 mg/kg dose did not show any significant decrease in pain-related behavior.

A dose–response curve of ibuprofen administered i.p. was constructed using the pain-related behavior in phase 2a of the formalin test against logarithmic transformed ibuprofen i.p. doses and a sigmoidal fit was applied. The mean IC_50_ was 69 mg/kg.

### Effect of different concentrations of ibuprofen administered in drinking water on formalin-induced pain-related behavior in mice

**Figure 2:**
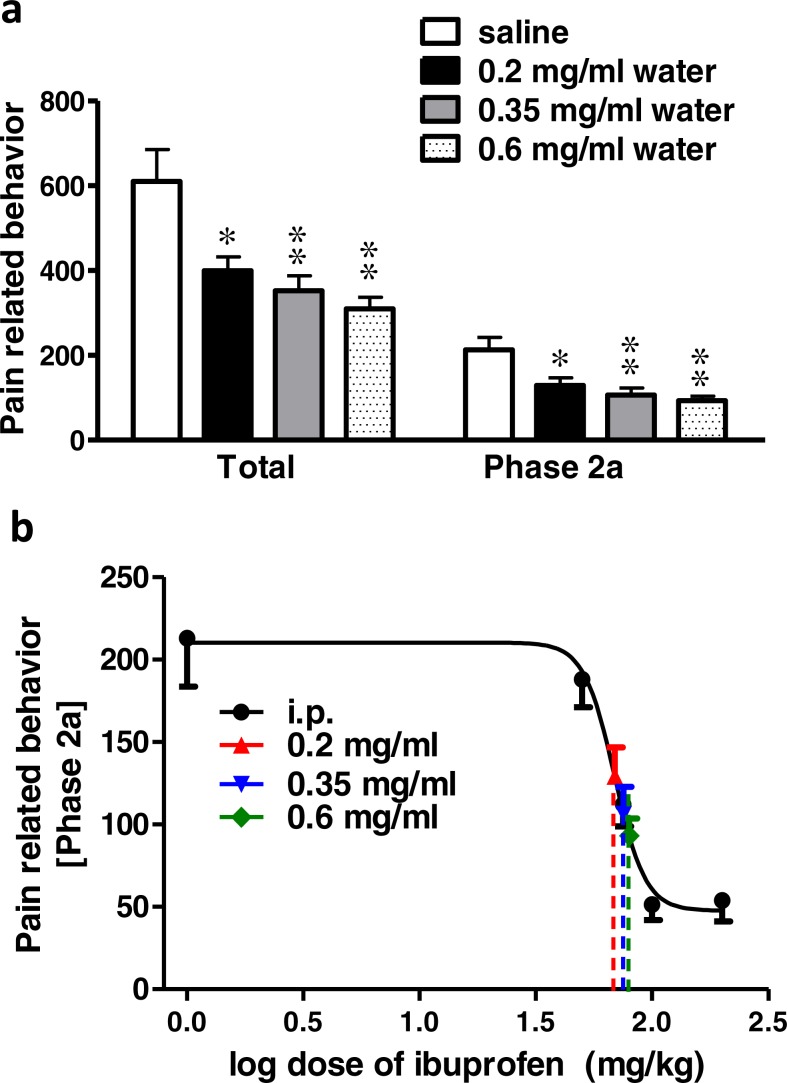
Effect of different concentrations of ibuprofen administered in drinking water on formalin-induced pain-related behavior in mice and equivalent i.p. doses. (A) Effect of different concentrations of ibuprofen administered in drinking water on formalin-induced pain-related behavior in mice. Data are presented as mean ± S.E.M. Each column represents the sum of pain-related behavior. The total represent the 60 min observation period. Phase 2a represents the time between the 20^th^ and the 40^th^ min. ^∗^ indicates *p* < 0.05 and ^∗∗^ indicates *p* < 0.01 compared to saline injected group (one way ANOVA, followed by Dunnett’s multiple comparison test). (B) Dose-response curve of ibuprofen administered i.p. The curve was constructed using the pain-related behavior in phase 2a of the formalin test against logarithmic transformed ibuprofen i.p. doses of 50, 75, 100 and 200 mg/kg b.w. A sigmoid fit was applied and equivalent i.p. doses to different doses of ibuprofen administered in drinking water were interpolated.

Ibuprofen administered in drinking water in different concentrations (0.2, 0.35 and 0.6 mg/ml), two days before the formalin test, resulted in a significant decrease in pain-related behavior. As seen in Fig. 2A 0.2, 0.35 and 0.6 mg/ml induced a significant decrease in both the total pain-related behavior and phase 2a (*p* < 0.05 for 0.2 mg/ml and *p* < 0.01 for 0.35 and 0.6 mg/ml; One-way ANOVA followed by Dunnett’s multiple comparison posttest).

**Figure 3:**
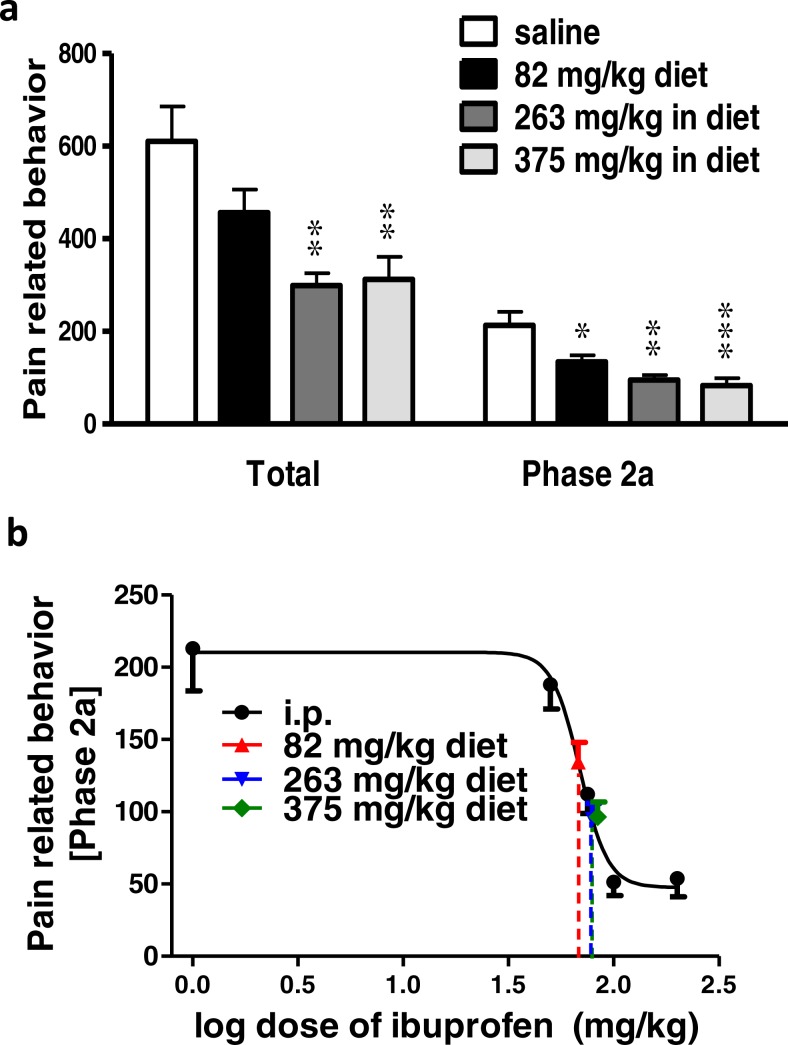
Effect of different doses of ibuprofen supplemented in diet on formalin-induced pain-related behavior in mice and equivalent i.p. doses. (A) Effect of different doses of ibuprofen supplemented in diet on formalin-induced pain-related behavior in mice. Data are presented as mean ± S.E.M. Each column represents the sum of pain-related behavior. The total represents the 60 min observation period. Phase 2a represents the time between the 20^th^ and the 40^th^ min. ^∗^ indicates *p* < 0.05, ^∗∗^ indicates *p* < 0.01 and ^∗∗∗^ indicates *p* < 0.001 compared to saline injected group (one way ANOVA, followed by Dunnett’s multiple comparison test). (B) The dose-response curve of ibuprofen administered i.p. The curve was constructed using the pain-related behavior in phase 2a of the formalin test against logarithmic transformed ibuprofen i.p. doses of 50, 75, 100 and 200 mg/kg b.w. Equivalent i.p. doses to different doses of ibuprofen administered in diet were interpolated.

Using the sigmoidal fit of the dose–response curve of ibuprofen administered i.p. and responses to different doses of ibuprofen administered in drinking water, equivalent i.p. doses were interpolated (Fig. 2B).

Concentrations of 0.2, 0.35 and 0.6 mg/ml in drinking water were found to be equivalent to 73.6 ± 5.2 mg/kg, 80 ± 18.6 mg/kg and 85.5 ± 20.9 mg/kg i.p., respectively.

### Effect of different doses of ibuprofen supplemented in diet on formalin-induced pain-related behavior in mice

Ibuprofen supplemented in diet in different doses (82, 263, 375 mg/kg diet), two days before the formalin test, resulted in significant decrease in pain-related behavior.

Ibuprofen supplemented in diet at doses of 263 and 375 mg/kg diet induced a significant decrease in total pain-related behavior (*p* < 0.01; One-way ANOVA followed by Dunnett’s multiple comparison posttest). Both doses also induced a significant decrease in pain-related behavior in phase 2a (*p* < 0.01 for 263 mg/kg diet and *p* < 0.001 for 375 mg/kg diet; One-way ANOVA followed by Dunnett’s multiple comparison posttest; Fig. 3A). As for the dose of 82 mg/kg diet, it induced a significant decrease in pain-related behavior in phase 2a only (*p* < 0.05; One-way ANOVA followed by Dunnett’s multiple comparison posttest).

Responses to different doses of ibuprofen supplemented in diet were used to interpolate the equivalent i.p. doses. Figure 3B presents the sigmoid fit of dose–response curve of ibuprofen, administered i.p. The curve was constructed using the pain-related behavior in phase 2a of the formalin test. Doses of 82, 263 and 375 mg/kg diet were found to be equivalent to 67.6 ± 9.3 mg/kg, 79.1 ± 11.7 mg/kg and 83.8 ± 11.4 mg/kg i.p., respectively.

### Effect of different doses of ibuprofen injected i.p. on surgical incision-induced tactile allodynia

**Figure 4:**
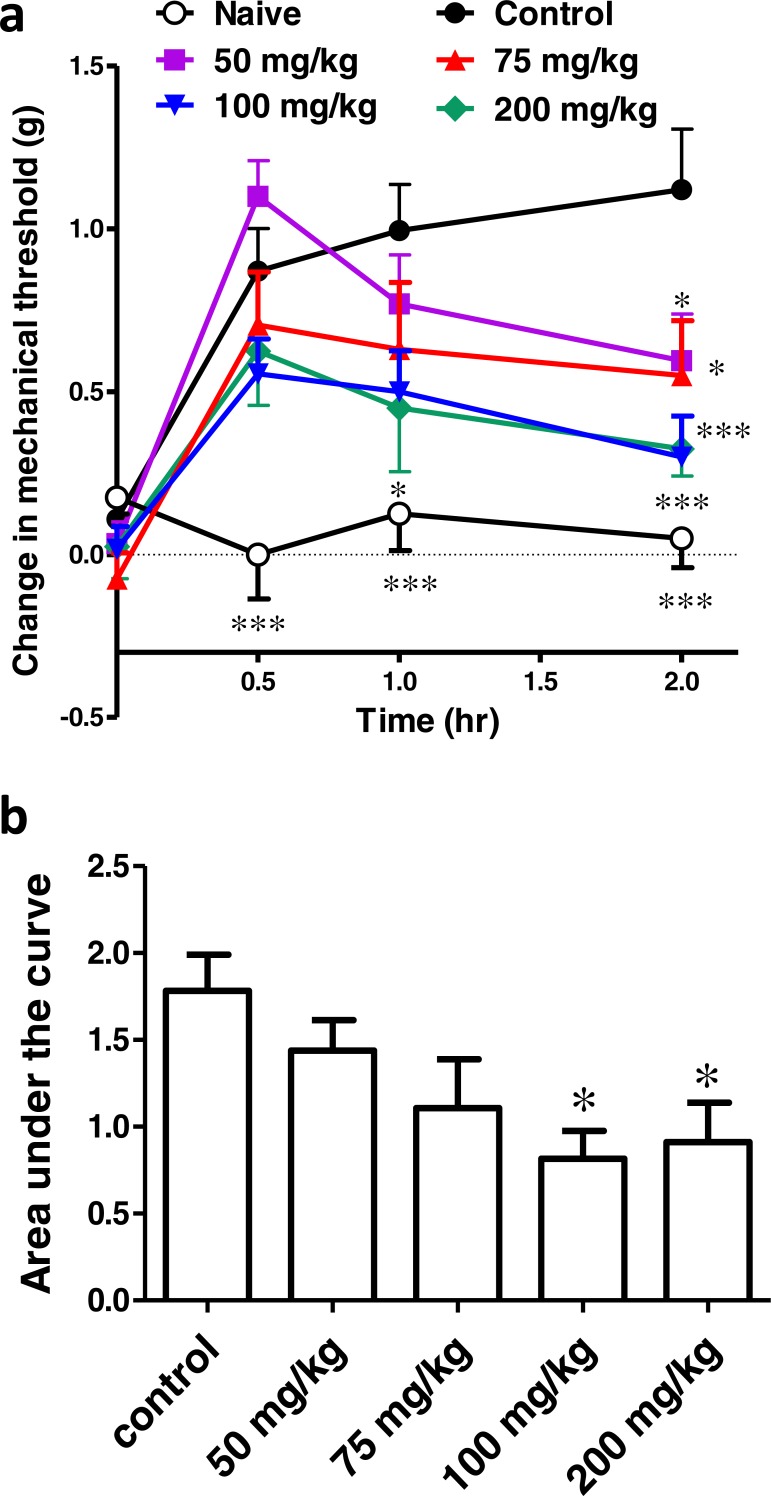
Effect of different doses of ibuprofen, administered i.p., on surgical incision-induced tactile allodynia in mice. (A) Line graph representing the difference in 60% mechanical threshold between the incised and non-incised paws in different groups at different time points (the change in mechanical threshold). Ibuprofen was administered 30 min before the incision at doses of 50, 75, 100 & 200 mg/kg i.p. Data are presented as mean ± S.E.M., where ^∗^ indicates *p* < 0.05 and ^∗∗∗^ indicates *p* < 0.001, compared to the control group (two-way ANOVA followed by Bonferroni’s post-test; *n* = 8). (B) Area under the curve of the upper figure (the area under the change in mechanical threshold-time curve; AUC_0–2h_), ^∗^ indicates *p* < 0.05 compared to the control group (One-way ANOVA followed by Dunnett’s multiple comparison test).

Surgical incision resulted in significant tactile allodynia, and ibuprofen injected i.p. in different doses (50, 75, 100 & 200 mg/kg), 30 min before incision, significantly reduced the development of tactile allodynia. Repeated-measure two-way ANOVA showed a significant effect of treatment (*p* = 0.0002; *F*(5, 42) = 6.42), significant effect of time (*p* < 0.0001; *F*(3, 126) = 39.73), and a significant interaction between time and treatment (*p* < 0.0001; *F*(15, 126) = 4.2). While there was no significant difference at the baseline among the different groups (*p* > 0.05; One-way ANOVA; Fig. 4A), the naive group was significantly different from the control group at all time points (*p* < 0.001, One-way ANOVA followed by Dunnett’s multiple comparison posttest).

Ibuprofen at doses of 50, 75 and 100 mg/kg showed a significant antiallodynic effect when compared to the control group, only at the 2 h time point (*p* < 0.05 for the 50 and 75 mg/kg and *p* < 0.001 for the 100 mg/kg; One-way ANOVA followed by Dunnett’s multiple comparison posttest). On the other hand, ibuprofen at a dose of 200 mg/kg showed a significant difference from the control group at 1 h (*p* < 0.05) and 2 h (*p* < 0.001; One-way ANOVA followed by Dunnett’s multiple comparison posttest; Fig. 4A).

The area under the ‘change in mechanical threshold-time’ curves (AUC_0–2h_), was significantly lower at the 100 and 200 mg/kg doses compared to the control group (*p* < 0.05, One-way ANOVA followed by Dunnett’s multiple comparison test; Fig. 4B).

A dose–response curve of ibuprofen, administered i.p. was constructed by plotting the area under the ‘change in mechanical threshold-time’ curves (AUC_0–2h_) against logarithmic-transformed ibuprofen i.p. doses and a sigmoidal fit was applied. The mean IC_50_ was 57 mg/kg.

### Effect of different concentrations of ibuprofen administered in drinking water on surgical incision-induced tactile allodynia

**Figure 5:**
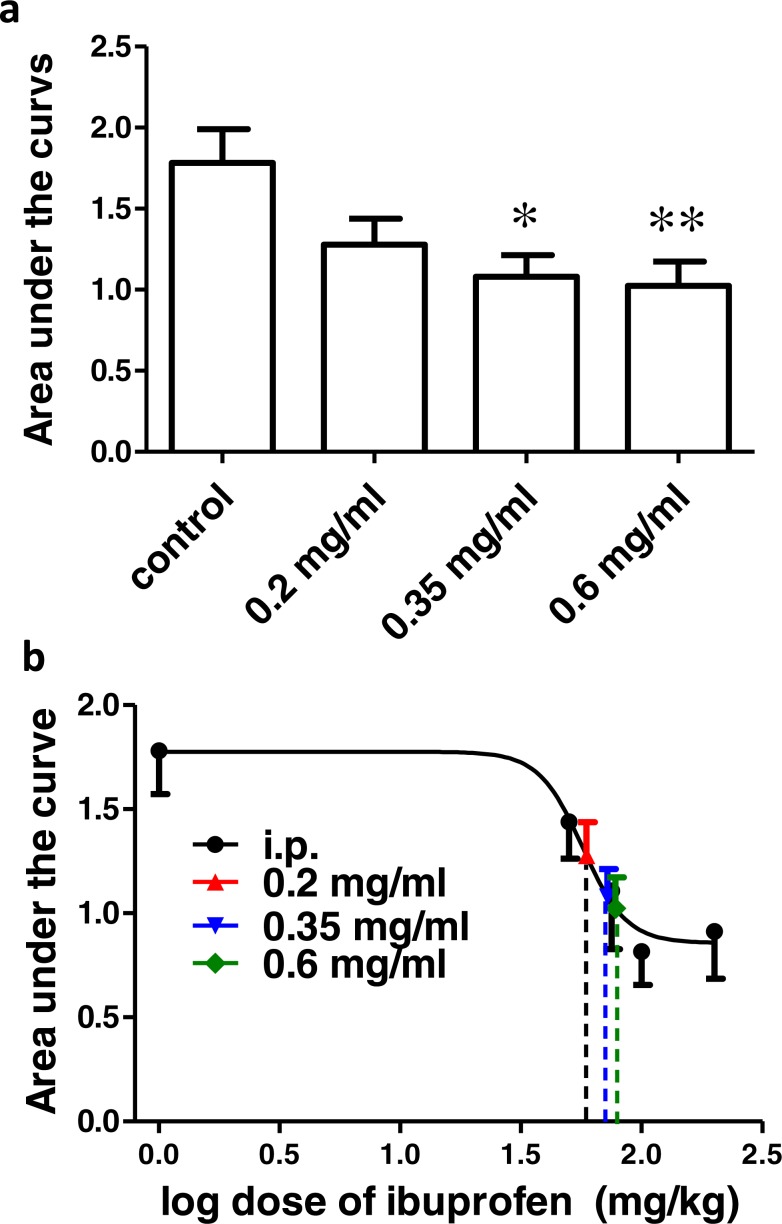
Effect of different concentrations of ibuprofen administered in drinking water on surgical incision-induced tactile allodynia in mice. (A) Area under the change in mechanical threshold-time curve (AUC_0–2h_) in mice treated with ibuprofen administered in drinking water, 2 days before the incision, at concentrations of 0.2, 0.35 & 0.6 mg/ml. Data are presented as mean ± S.E.M., ^∗^ indicates *p* < 0.05, ^∗∗^ indicates *p* < 0.01 compared to the control group (One-way ANOVA followed by Dunnett’s multiple comparison test; *n* = 8). (B) Dose-response curve of ibuprofen administered i.p. The curve was constructed using the area under the change in mechanical threshold time curves (AUC_0-2h_) against logarithmic transformed ibuprofen i.p. doses of 50, 75, 100 and 200 mg/kg b.w. A sigmoid fit was applied and mean equivalent i.p. doses to different doses of ibuprofen administered in drinking water were interpolated.

Ibuprofen was given in drinking water in different concentrations (0.2, 0.35, 0.6 mg/ml), two days before the incision. AUC_0−2*h*_ was significantly lower in the 0.35 and 0.6 mg/ml concentrations, compared to the control group (*p* < 0.05 and *p* < 0.01, respectively, one-way ANOVA followed by Dunnett’s multiple comparison test; Fig. 5A).

Figure 5B shows the dose–response curve of ibuprofen, administered i.p. Responses to different doses of ibuprofen administered in drinking water were used to interpolate the equivalent i.p. doses. The concentrations of 0.2, 0.35 and 0.6 mg/ml in drinking water were found to be equivalent to 58.9 ± 31.7 mg/kg, 72.2 ± 31.1 mg/kg and 77.8 ± 26.7 mg/kg i.p., respectively.

### Effect of different doses of ibuprofen supplemented in diet on surgical incision-induced tactile allodynia

**Figure 6:**
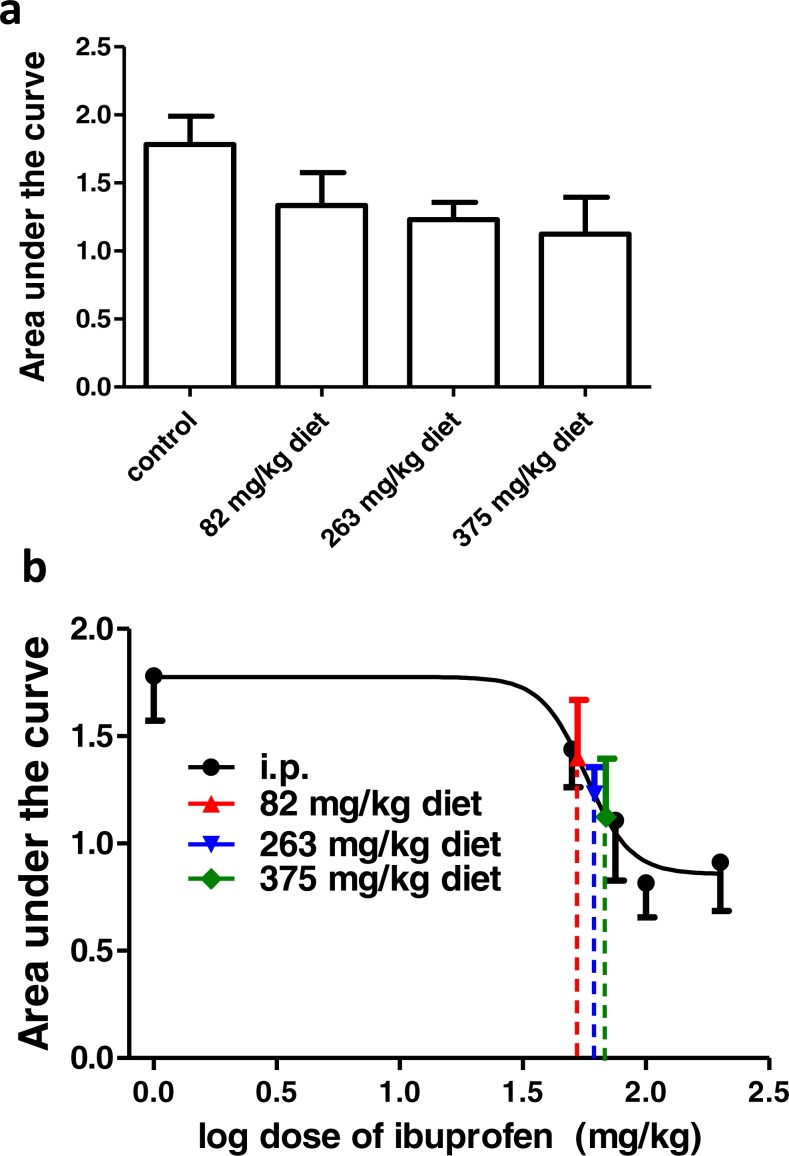
Effect of different doses of ibuprofen supplemented in diet on surgical incision-induced tactile allodynia. (A) Area under the curve of the change in mechanical threshold-time curve (AUC_0-2h_) in mice treated with ibuprofen administered in diet, 2 days before the incision at concentrations of 82, 263 & 375 mg/kg diet. Data are presented as mean ± S.E.M., *n* = 8/group. (B) Dose-response curve of ibuprofen administered i.p. The curve was constructed using the area under the change in mechanical threshold-time curves (AUC_0-2h_) against logarithmic transformed ibuprofen i.p. doses of 50, 75, 100 and 200 mg/kg b.w. A sigmoid fit was applied and mean equivalent i.p. doses to different doses of ibuprofen administered in diet were interpolated.

Ibuprofen was given in diet in different doses (82, 263, 375 mg/kg diet), two days before surgical incision. AUC_0–2h_ was not significantly different in any of the groups, compared to the control group (*p* > 0.05, One-way ANOVA followed by Dunnett’s multiple comparison test; Fig. 6A).

Using the sigmoidal fit for the dose–response curve of ibuprofen administered i.p., 82, 263 and 375 mg/kg diet were equivalent to 52.7 ± 14.2 mg/kg, 61.6 ± 22.9 mg/kg and 68.6 ± 32.6 mg/kg i.p., respectively (Fig. 6B).

### Effect of different doses of ibuprofen on serum TXB_2_

**Figure 7:**
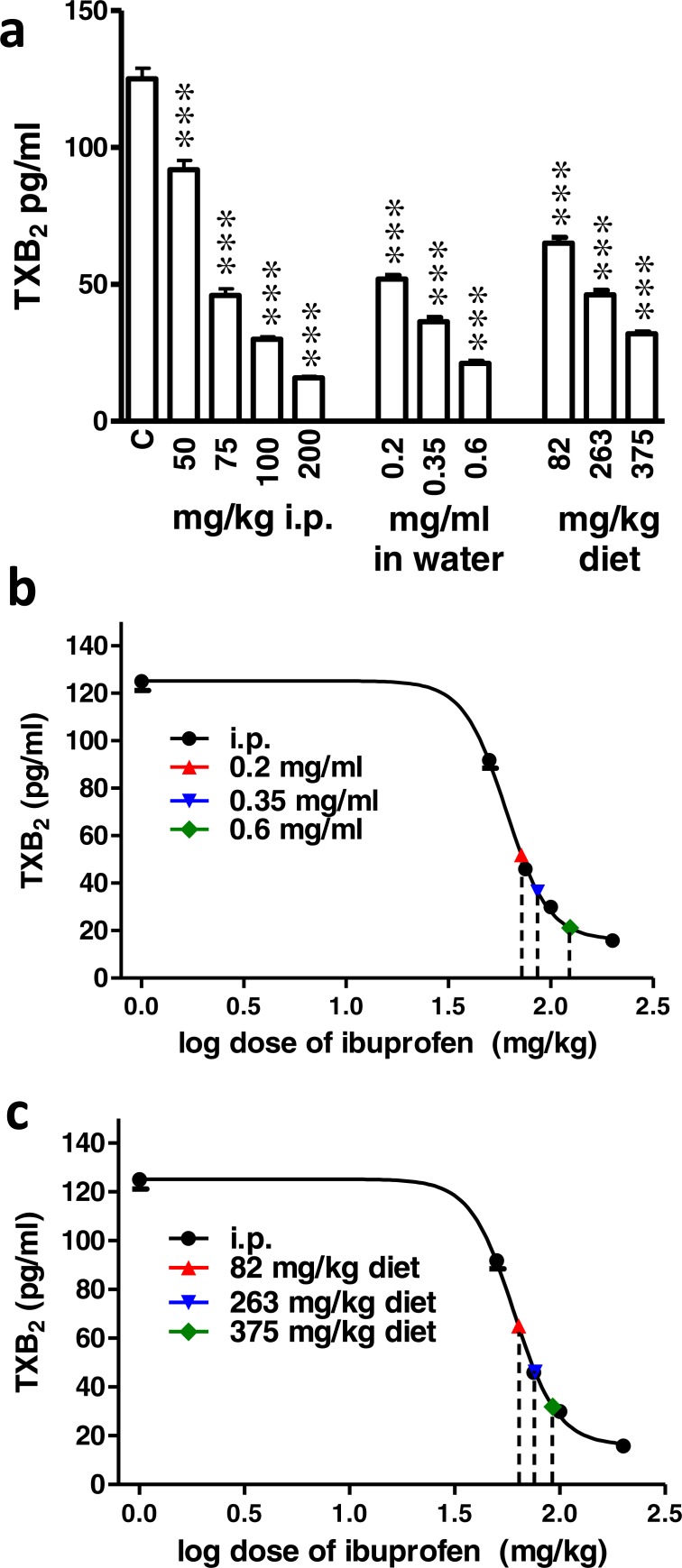
Effect of different doses of ibuprofen on serum TXB_2_. (A) Serum TXB_2_ concentration in the control (C), i.p. ibuprofen treated groups, ibuprofen supplemented in drinking water and in diet groups. Ibuprofen was administered i.p. 2 h before sample collection or in drinking water or diet for 2 days before the sample collection. Each column represents the mean ± S.E.M., where ^∗∗∗^ indicates *p* < 0.001 compared to the control group (One-way ANOVA followed by Dunnett’s multiple comparison posttest; *n* = 6). (B & C) A dose-response curve of serum TXB_2_ concentrations was constructed against logarithmic transformed i.p. doses of ibuprofen (50, 75, 100 and 200 mg/kg b.w.). A sigmoidal fit was applied and equivalent i.p. doses to different doses of ibuprofen administered in drinking water (B) or in diet (C) were interpolated.

Ibuprofen administered i.p. in different doses (50, 75, 100 & 200 mg/kg) two hours before blood collection caused a significant dose dependent decrease in the TXB_2_ concentration compared to the control group (*p* < 0.001; One-way ANOVA followed by Dunnett’s multiple comparison posttest; Fig. 7A). When supplemented in drinking water for two days before blood samples collection, ibuprofen in different concentrations (0.2, 0.35, 0.6 mg/ml) caused a significant dose dependent decrease in the TXB_2_ concentration compared to the control group (*p* < 0.001; One-way ANOVA followed by Dunnett’s multiple comparison posttest; Fig. 7A). Similarly, when supplemented in diet for two days before samples collection, ibuprofen in different doses (82, 263 & 375 mg/kg diet) caused a significant decrease in the TXB_2_ concentration compared to the control group (*p* < 0.001; One-way ANOVA followed by Dunnett’s multiple comparison posttest; Fig. 7A).

Figures 7B and 7C show the sigmoidal fit of the dose–response curve of serum TXB_2_ levels in response to i.p. administered ibuprofen. The mean IC_50_ was 60.5 mg/kg. Comparing the IC_50_ of ibuprofen in the three tests did not show a significant difference (*p* = 0.99, one way ANOVA).

Ibuprofen administered in drinking water at a concentration of 0.2, 0.35 and 0.6 mg/ml were found to be equivalent to 71.8 ± 2.3 mg/kg, 86.4 ± 5 mg/kg and 125.8 ± 10.6 mg/kg i.p., respectively (Fig. 7B). On the other hand, ibuprofen administered in diet, 82, 263 and 375 mg/kg diet were found to be equivalent to 63.6 ± 3 mg/kg, 76.3 ± 3.4 mg/kg and 92.5 ± 2.8 mg/kg i.p., respectively (Fig. 7C).

**Figure 8:**
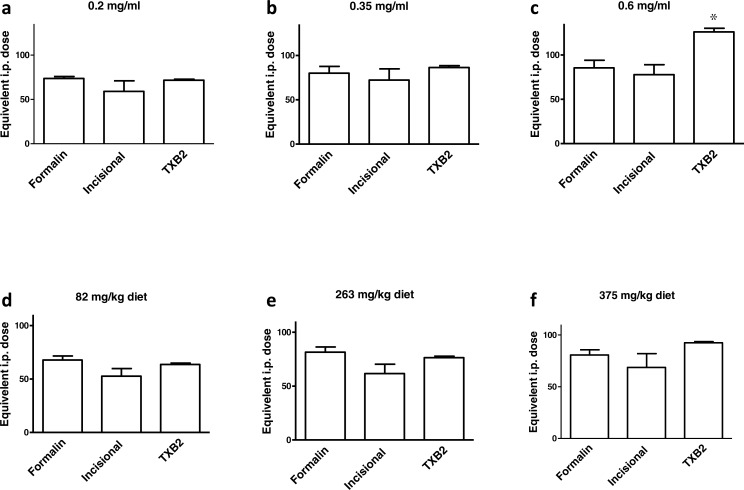
A summary of the results. Equivalent i.p. doses to different doses of ibuprofen administered in drinking water or in diet in the 3 experimental settings. Data are presented as mean ± S.E.M. ^∗^ indicates *p* < 0.05 compared to equivalent doses in the formalin test and *p* < 0.01 compared to equivalent doses in the incisional pain model, One way ANOVA, followed by Bonferroni’s multiple comparison test.

A summary of the equivalent i.p. doses in mg/kg b.w. for ibuprofen administered in diet or in drinking water in the behavioral and biochemical experiments performed is shown in Fig. 8. There was no significant difference between the equivalent i.p. doses in the three experiments in any of the doses tested in the present work, with the exception of the 0.6 mg/ml concentration. The equivalent i.p. dose for the 0.6 mg/ml concentration in the TXB_2_ assay was significantly higher than those seen in the formalin test (*p* > 0.05) or in the incisional pain test (*p* < 0.01, one-way ANOVA followed by Bonferroni’s multiple comparison test).

### Water consumption in mice

**Figure 9:**
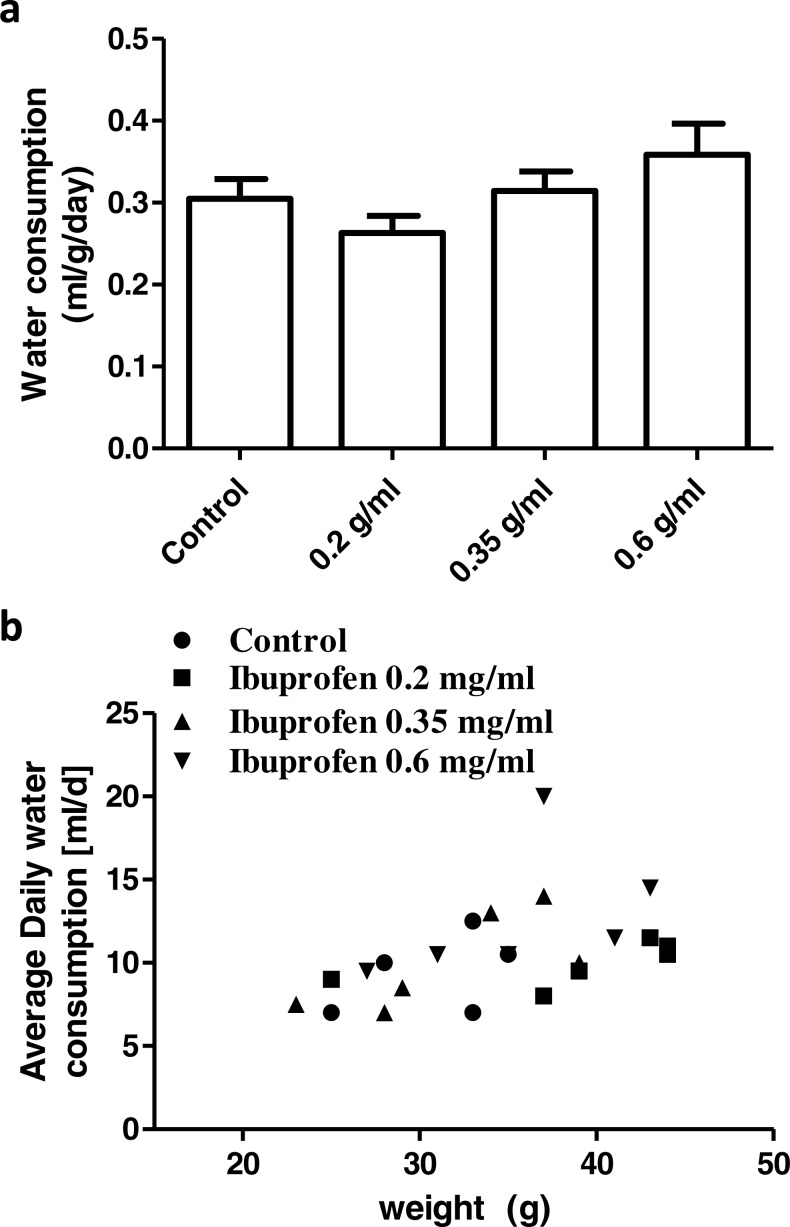
Daily water consumption. (A) Weight corrected average daily water consumption in control group and in groups treated with ibuprofen in drinking water. Each column represents the mean ± S.E.M. (*n* = 6). (B) Scatter plot correlating the average daily water consumption to the mice body weight (*r* = 0.48; *p* = 0.018; Pearson correlation).

None of the concentrations of ibuprofen in drinking water (0.2, 0.35, 0.6 mg/ml) caused a significant difference in the average daily water consumption, compared to the control group (*P* > 0.05; One-way ANOVA followed by Dunnett’s multiple comparison posttest; Fig. 9A). There was a significant correlation between the average daily water consumption and the mice body weight (*r* = 0.48; *p* = 0.018; Pearson correlation; Fig. 9B).

## Discussion

It is important, when administering drugs to experimental animals, to make appropriate decisions on several factors; including the dose, the route of administration and the solvent. Odor and taste of the compound should also be considered (Hayward et al., 2006). The choice of ibuprofen sodium salt, in the present study, was based on the above-mentioned considerations. It is soluble in water and therefore can be dissolved in drinking water. It does not affect taste or odor and thus does not affect water consumption in mice. This is apparent by the insignificant difference in water consumption among different groups of mice, shown here.

The water and food consumption varies among strains (Bachmanov et al., 2002), and might differentially affect drug intake, if administered in drinking water or in diet. They are also affected by the weight of mice. This is ideal in the case of drug therapy, given that doses are always calculated in relation to body weight. In the present study, there was a significant correlation between water consumption and body weight. However, we could not accurately evaluate the food consumption and therefore could not assume a similar relationship, yet it is well documented in the literature (Bachmanov et al., 2002).

Experiments were performed after two days of ibuprofen administration in diet or in drinking water, since the half-life of ibuprofen is 2.5 h and it follows linear kinetics (Brocks & Jamali, 1999; Graham & Williams, 2004). Therefore, two days are far beyond the time needed to achieve a steady-state concentration.

In order to construct the appropriate dose–response curve here, ibuprofen was given i.p. in 4 doses (50, 75, 100, 200 mg/kg). 200 mg/kg was the maximal tolerable dose in mice. A higher dose (300 mg/kg) caused the sudden death. This is in accordance with Adams et al. (1969), who reported an approximate LD_50_ of 320 mg/kg for ibuprofen administered i.p. in the mouse. Given the 30 mg/kg was found to be ineffective in the incisional pain model in mice (Saad et al., 2016), we started the dose response curve with 50 mg/kg, which is equivalent to 243.9 mg in humans.

In constructing a dose–response curve for the formalin test, phase 2a was chosen. It is known that the effect of COX inhibitors, including ibuprofen, is most evident during phase 2a (Malmberg & Yaksh, 1995). Even though phase 2a is the most sensitive phase of the formalin test to COX inhibitors, ibuprofen in high doses significantly affected phase 1, too. This is in agreement with Malmberg & Yaksh (1995).

On the other hand, in the surgical pain model, the area under the ‘change in mechanical threshold-time’ curve was used in order to reflect the analgesic effect of ibuprofen throughout the first 2 h. The post-incisional pain is most prominent at the first 2 h and therefore, the analgesic effect of ibuprofen can be better observed. Indeed the 2 h time point is the one, at which all the range of ibuprofen doses administered i.p. demonstrated an antiallodynic effect. This is expected in light of the consensus that the antinociceptive action of ibuprofen is more precisely described as antihyperalgesic rather than as analgesic (Burian & Geisslinger, 2005). In the same context, serum TXB_2_ was chosen to construct the dose–response curve. It is possible to estimate TXB_2_ inhibition both in plasma and in serum. However, incubating blood samples in a 37 °C water bath for 1 h before centrifugation results in exceptionally higher TXB_2_ levels in blood (Brideau et al., 1996; Patrignani et al., 1997). Thus, a more pronounced inhibitory effect of COX inhibitors can be seen, when TXB_2_ is estimated in serum (Good et al., 2015).

IC_50_ could be calculated from the dose–response curves, to show that there was no significant difference among the three tests. Given that in intact cells, IC_50_ of ibuprofen is 4.85 pmol/dl^3^ for COX-1 and 72.6 pmol/dl^3^ for COX-2, with a COX-2:COX-1 selectivity ratio of 15 (Frolich, 1997), our results suggest a major role for COX-1 in the formalin test and the incisional pain model at the time frame described. This is in accordance with earlier reports that that COX-1 activity is increased during the early phases of inflammatory pain (Wallace et al., 1998) and that the increase in spinal COX-2 mRNA occurs within 2–6 h after inflammation (Beiche et al., 1996). It is known that both COX-1 and COX-2 are independently involved in the generation of PGE_2_ and hence in central sensitization (Islam et al., 2016).

Dose–response data are usually displayed on a semilogarithmic scale to transform the hyperbolic curve into a sigmoid curve with linear mid-portion (Von Zastrow, 2015). In the present study, both the behavioral and biochemical dose–response curves showed a sigmoidal pattern with a linear mid-portion. Most of the points of the ibuprofen supplemented in drinking water or in diet lay within this linear mid-portion of the curve. It is noticeable that increasing the dose of ibuprofen administered in drinking water or in food here, did not result in an equivalent increase in the pharmacological effect. It does not seem possible, therefore, to predict the response of doses administered in drinking water or in food mathematically based on the dose–response curve of i.p. administered ibuprofen.

It is reported that 0.2 mg/ml of ibuprofen in drinking water provided a daily dose of approximately 40 mg/kg ibuprofen in C57BL/6J mice with average daily water consumption of 5–6 ml (Hayes et al., 2000). In the present study, Swiss albino mice consumed an average of 9.6 ml/day. When taking the body weight into consideration, the average daily dose would approximately be 60 mg/kg. However, judging by pharmacodynamic responses, 0.2 mg/ml ibuprofen in drinking water, in the present work, induced an effect equivalent to that of approximately 70 mg/kg b.w., i.p., in the formalin test and the TXB_2_ inhibitory effect.

Jalbert & Castonguay (1992) reported that ibuprofen supplemented in diet at a dose of 263 mg/kg provided a daily dose of approximately 25 mg/kg b.w. in adult A/J mice. On the other hand, Yan et al. (2003) reported that ibuprofen supplemented in diet at a dose of 375 mg/kg provided a daily dose of approximately 62.5 mg/kg b.w. in elderly APP transgenic Tg2576 mice. Both studies used average estimated food consumption to calculate the approximate daily dose. In the present work, ibuprofen supplemented in diet at a dose of 263 mg/kg induced an effect equivalent to that of i.p. doses ranging between 61 and 79 mg/kg b.w. On the other hand, when ibuprofen was supplemented in diet at a dose of 375 mg/kg, it induced an effect equivalent to that of i.p. doses ranging between 68 and 92 mg/kg b.w. These equivalent doses are based on the three tests performed here. Strain differences may partly account for these differences.

It is expected that response to drugs administered orally would vary quantitatively from those administered parenterally. In this regards, the difference in response to oral versus intraperitoneal doses of ibuprofen was reported earlier by Adams et al. (1969). They reported that LD_50_ for ibuprofen in mice was 800 mg/kg, when administered orally; compared to 320 mg/kg, when administered i.p. It is expected that pharmacokinetic aspects may result in this variability. However, the pharmacokinetics of i.p. administered drugs are close to those of orally administered drugs, because they are both absorbed into the mesenteric vessels (Ludvig et al., 2009). Therefore, ibuprofen administered by either route would be subjected to hepatic first-pass metabolism. Further, the bioavailability of ibuprofen sodium is approximately 100% (Martin et al., 1990; Sorgel et al., 2005).

There is an obvious link between the pharmacokinetics of ibuprofen and its pharmacodynamics, as ibuprofen (racemate) exist in an equal mixture of R-ibuprofen (not prostaglandin synthesis inhibiting) and S-ibuprofen (prostaglandin synthesis inhibiting; Bonabello et al., 2003). Unidirectional metabolic chiral inversion of the inactive R-enantiomer to the S-form occurs in the intestinal tract and liver after oral absorption (Jamali et al., 1992). This conversion of racemic ibuprofen to active S-isomer may play a role in the variability of analgesia (Laska et al., 1986). Systemic inversion contributes to the inversion process. S-ibuprofen is formed after intravenous administration of R-ibuprofen (Cheng et al., 1994). Several stress-related factors, including acute surgical pain, affect gastrointestinal functions; and are therefore proposed to reduce gastric absorption of ibuprofen. The bioconversion of the inactive (R−) enantiomer to the active (S+) enantiomer may also be affected by such stress-related factors (Jamali & Kunz-Dober, 1999).

Stress conditions may be considered in the present study due to pain and social isolation prior to pain induction, which may therefore explain the disproportionate increase in the response to orally administered ibuprofen with increasing the dose unlike the i.p. administered drug. In this regard, equivalent i.p. doses in the surgical pain model were lower than those in the other two tests, though insignificantly. This may be due to the exposure of the mice in this model to ether anesthesia, which delays gastric emptying, affecting absorption in the small intestine (Reynell & Spray, 1957). Furthermore, measurement of TXB_2_ was performed in serum, where mice were not subjected to previous isolation, stress, or surgery. The equivalent i.p. dose was significantly higher in case of TXB_2_ assay, when ibuprofen was administered in drinking water at concentration of 0.6 mg/ml, in the present study. The higher effect cannot be ascribed to exceeding water consumption, since there was no significant difference in water consumption among different groups. However, this was not seen in the other concentrations. It has been reported that there are difficulties encountered in the field of measuring response to aspirin as regard TXB_2_ level (Good et al., 2015); and there has been poor correlations between assays of aspirin responsiveness (Good et al., 2015; Snoep et al., 2007; Sofi et al., 2008). These may also explain the different equivalent doses seen with TXB_2_ measurement in the present study.

It is expected that the pharmcodynamic properties of a drug are affected by the rate of its administration, or in other words the rate of its absorption (Kleinbloesem et al., 1987). Therefore, when ibuprofen is supplemented in drinking water or in diet and its administration is continuous, different pharmacodynamic effects may be observed. The smaller and more frequent the doses in continuous administration, the smaller the swings in concentration (Rang et al., 2016). It therefore lacks the early rise in plasma concentration level (*C*_max_) achieved with the single i.p. large dose. However, estimation of ibuprofen plasma level is required to confirm this suggestion. In addition, repeating the standard experiments using oral gavage instead of i.p., route may help explain the present results. Both are to be further studied.

## Conclusion

The increment in pharmacological effects of different doses of continuously administered ibuprofen in drinking water or diet do not parallel those of i.p. administered ibuprofen. It is therefore difficult to assume these equivalent doses based merely on mathematical calculations.

##  Supplemental Information

10.7717/peerj.2239/supp-1 Supplemental Information 1 Raw data

## Additional Information and Declarations

### Data Availability

The following information was supplied regarding data availability: The raw data has been supplied as Data S1.
